# Congress News

**Published:** 2017

**Authors:** PA Brink

Khartoum, what a location for the joint congress of PASCAR, the Sudan Heart Society and the Pan-African Interventional Cardiology Course (PAFCIC)! The PAFCIC (http://pafcic.org/) took place alongside the main congress and presented the practicalities of interventional procedures. The African summit of the World Heart Federation was also held in proximity to the congress (proceedings documented at https://www.world-heart-federation.org/whf-african-summit/).

The joint congress was housed in the Friendship Hall, in Khartoum, next to the Blue Nile, a kilometre or so from the confluence of the two Nile rivers. The opening by General Bakris Hassan Saleh, first vice-president of the republic and national prime minister of Sudan was magnificent. Proceedings were in Arabic, but one could follow a translation into English on small headsets.

After a few words from Prof Bongani Mayosi, president of PASCAR, delegates were introduced to the guest speaker, Prof AA Gehani from the Heart Centre, Cornell Medical Centre, Qatar. Prof Gehani advanced a well-supported hypothesis that Ibn-Nafis, a 13th century Arabic scholar, described the pulmonary circulatory system about four centuries before William Harvey did. He also explained how Harvey may have read this, having had access to Arabic sources that had been translated into Latin. This theory is not new and appears in a letter to the Cardiovascular Journal of Africa in 2009; 20(5): 299.

The main themes of the congress were around diseases endemic to Africa: rheumatic heart disease, hypertension and heart failure, but a fair share of the programme revolved around ischaemic heart disease and related interventions. Some space was also allotted to congenital and arrhythmic heart disease.

Presentations were almost along the lines of taking stock: ‘What cardiovascular disease do we see? How frequent is it? What is available to diagnose and manage it? How do we compare among each other and compared to developed countries, and is what we do appropriate for Africa?’ For example, Bongani Mayosi, stated that rheumatic heart disease is still endemic in all parts of Africa, major parts of Asia and in some pockets elsewhere in the world. He explained how some countries have managed to decrease its prevalence through increasing awareness and prevention. Eighty per cent of premature cardiovascular disease and death occurs in developing countries, a very significant proportion of which is Africa.

Karen Sliwa addressed the issue of whether Africa can meet the 25 × 25 goal of the United Nations to achieve a 25% reduction in premature mortality from cardiovascular disease by 2025. Others speakers, Ibtisam Ali, Anastase Dzudie, Albertino Damasceno, Elijah, Salim Yusuf and Gerald Yonga talked about how to address the major risk factors, such as hypertension, smoking and others. A major issue is the cost and quality of medication and the payment thereof. Three ministers of health, who are important for formulating and implementing public health policies with regard to smoking, sugar consumption, hypertension and more, unfortunately did not put in an appearance.

The work of Salim Yusuf from Canada is fascinating. His group has been studying cardiovascular disease on a global scale by asking simple questions such as: ‘Is the incidence and prevalence of events the same? If a myocardial infarction or stroke occurred, are the “risk” factors the same? Are the outcomes of events the same or different in different settings?’ On this last question, Yusuf asked, ‘If one finds oneself having chest pain in an area where primary percutaneous coronary intervention (PPCI) is not timeously available, if at all, and even appropriately given thrombolysis may not be available, what should one do?’ He ventured an idea for self-treatment; to carry an emergency ‘cocktail’ of aspirin, an ACE inhibitor, a β-blocker and a statin. This makes sense, even in Khartoum.

Ahmed Suliman gave an overview of the situation in Sudan. Even in Khartoum, with many private and some public cardiac catheterisation laboratories (CCL), you are likely to get timely PPCI only if you pay privately. In the public system, at primary care facilities, after first dealing with issues of payment, the window of opportunity will have passed, and even thrombolysis will not be done. According to Toure, in Niger there is no CCL. Some other countries are in a similar situation and Habib Gamra has compiled a very useful interventional map for Africa.

The cardiac surgery situation in Sudan is interesting. Besides the Sudanese facilities, elaborated on by Kamal Khoghali, there is also a major centre, the Salam Centre, funded by an NGO and expounded on by Alessandro Salvati, which undertakes surgery and follow up at no cost.

Representatives of both the Cardiovascular Journal of Africa and the Sudan Heart Journal (SHJ) had the opportunity of giving an overview. Siddiq Khalil, the editor of SHJ, gave an excellent review on the origin of the journal in 2011, its policies, growth and aims. He also spoke about the history of Sudan and the conquering of Sudan by General Horatio Kitchener in 1898. This is a name we in South Africa can identify with, thinking of the role Kitchener played in the Anglo–Boer war two years later.

The foreign delegates were well treated by a number of friendly young doctors who helped us into and out of Sudan, as well as getting us to where we needed to be. The evening outings to some excellent restaurants were very enjoyable despite the absence of alcohol.

